# Moses, money, and multiple-choice: The Moses illusion in a multiple-choice format with high incentives

**DOI:** 10.3758/s13421-020-01128-z

**Published:** 2020-12-21

**Authors:** Felix Speckmann, Christian Unkelbach

**Affiliations:** Social Cognition Center Cologne, University of Cologne, Richard-Strauss-Straße 2, 50931 Köln, Germany

**Keywords:** Moses illusion, Cognitive illusions, Incentivized responding, Response biases

## Abstract

When people answer the question “How many animals of each kind did Moses take on the Ark?”, they usually respond with “two,” although Moses does not appear in the biblical story of the Ark. We investigated this “Moses illusion” in a multiple-choice format and tested the influence of monetary incentives on the illusion’s strength. Thereby, we addressed the role of a cooperative communication context for the illusion’s emergence, as well as the role of participants’ motivation. In four experiments (total N = 914), we found that the Moses illusion persists in a multiple-choice format. As the multiple-choice format realizes a cooperative context in which the correct answer is always available, we exclude a cooperative context explanation for the illusion. Monetary incentives reduced the strength of the illusion. However, the reduction was numerically and statistically small. We thereby show that the illusion is not due to violations of cooperative communications, and not due to a lack of motivation. The multiple-choice approach will facilitate further research on the Moses illusion and the data provide additional evidence for the Moses illusion’s empirical robustness and constrain its theoretical explanations.

## Introduction

“How many animals of each kind did Moses take on the Ark?” Most people spontaneously respond “two.” This erroneous response often occurs even when people know that, according to the Bible, it was Noah and not Moses who took two kinds of each animal onto the Ark. Hence, the question cannot be answered, but people readily answer it nonetheless. Since its seminal examination by Erickson and Mattson (1981), this “Moses” illusion has become a robust classic.

Here, we investigate the illusion in a multiple-choice format in which the correct response (“can’t be answered”) is available and we make responses relevant because people may win or lose money for correct and incorrect responses. Thereby, we answer two research questions regarding the Moses illusion. First, people may believe that questions should be answerable in principle, following a norm of cooperative communication. As Grice (1975) delineated, people expect communication to be cooperative; that is, respondents expect that they should be able to answer a question. However, if the illusion persists in a multiple-choice format, then it is unlikely to be due to norms of cooperative communication. People might respond “two” because the response fits to the setup of animals and the ark in a cooperative communication setting. Yet, providing the correct “can’t be answered” response as an option in the response set creates a cooperative communication setting; that is, the solution is present and available. Second, respondents may simply not care for the correct response and provide a response that at least partially fits with the question. However, if the illusion persists with incentivized responses, then it is unlikely due to participants’ superficiality and lack of motivation. Conversely, participants might not show the illusion if responses have real monetary consequences. Rather, they might retrieve the fact that Noah, not Moses, is the Biblical character who built the Ark.

In the remainder, we provide a short overview of research on the Moses illusion. Based on this overview, we delineate our two research questions in more detail. Finally, we present four experimental studies that address these questions empirically.

### Previous research on the Moses illusion

To keep the terminology consistent across authors and experiments, we refer to “Moses” questions (“How many animals did *Moses*…”) as “distorted” questions and to the question from which they are derived as “undistorted” questions (“How many animals did *Noah*…”). We refer to erroneous responses to Moses questions (e.g., “two” to the Moses question) as “Moses” answers. We refer to the term that is changed between distorted and undistorted questions as the “critical” term (e.g., “Moses” vs. “Noah”). We refer to the presence of the illusion when participants provide significantly more Moses responses as can be expected by chance.

#### The Moses illusion

Erickson and Mattson (1981) first demonstrated what is now well-known as the Moses illusion. They provided participants with a set of questions and informed them that some questions might be distorted and unanswerable. Across four “Moses” questions, participants provided 52.3% Moses answers (i.e., about two out of four on average), despite possessing the relevant knowledge. In Study 2, the authors shifted the focus of the question to the question’s critical term by moving it to the sentence’s beginning (i.e., “Moses took two animals of each kind on the Ark”) and used a “true” or “false” judgment format. This reduced the illusion but did not eliminate it; participants still provided 26.5% Moses answers, despite possessing the relevant knowledge. In Study 3, the question’s critical term varied (e.g., “Moses,” “Adam,” “Abraham,” or “Nixon” for the Moses question). The critical terms differed in their phonological and semantic similarity to the undistorted terms, and higher semantic relation resulted more participants falling for the illusion. Names without semantic relations (e.g., “Nixon took two animals…”) eliminated the illusion (see also Van Oostendorp & Kok, 1990). For all remaining cases, participants still provided 52.3% Moses answers, despite possessing the relevant knowledge.

Further research substantiated these findings; the illusion partially depends on the direction of focus (Bredart & Modolo, 1988), higher semantic relatedness between the names used in the questions (e.g., Noah and Moses vs. Noah and Adam) increases the illusion (Van Oostendorp & De Mul, 1990), and phonetic relatedness (e.g., identical number of syllables, identical first vowel) increases the illusion (Shafto & MacKay, 2000). In addition, lower processing fluency decreases the illusion (Song & Schwarz, 2008), expertise decreases the illusion (Cantor & Marsh, 2017), and olfactory cues metaphorically related to suspicion decrease the illusion (Lee, Kim, & Schwarz, 2015). In addition, there seem to be interindividual differences in access to long-term memory knowledge as well as short-term memory capacity that influence the illusion (Hannon & Daneman, 2001).

Next, we address two explanations that fit with the presented evidence in more detail and which are most relevant for the present research question.

#### The cooperative communication explanation

Reder and Kusbit (1991) investigated cooperative response behavior (Exp. 1) as an explanation. They argued that participants might notice the distortion but choose to ignore it to cooperate with the experimenter. To test this explanation, they used a “literal” and a “gist” condition. The literal condition instructed participants to answer questions literally, requiring the answer “can’t say” to Moses questions. The gist condition instructed participants to ignore minor inconsistencies, requiring the answer “two” to the Moses question.

Accordingly, if participants register the distortion and choose to ignore it, the literal condition should be easier, as the gist condition requires the step to ignore the distortion. However, in terms of participants’ errors and response latencies, the gist condition appeared to be easier. They thus concluded that the illusion does not appear due to participants cooperative behavior.

#### The partial-matching hypothesis

The partial-matching assumes that people only match certain parts of sentences to relevant memory structures when answering the questions (Reder & Cleeremans, 1990). In their study, Reder and Cleeremans also used a literal and a gist condition (see above). They argued that the gist condition is more suited for a partial matching strategy whereas the literal condition is more suited for a complete matching strategy. Based on participants’ superior performance in the gist condition, the authors concluded that the illusion follows from partial-matching. This argument is supported by the finding that the more terms within the distorted question match with the undistorted question, the stronger the illusion becomes. They further argued that the default processing mode aims for reduction effort (p. 248), and therefore, a partial match suffices to trigger a Moses response (see also Kamas et al., 1996; Park & Reder, 2004).

### The present research

We investigated two aspects of the Moses illusion that follow from the two presented explanations. First, we wanted to examine again the cooperative communication explanation. As discussed, Song and Schwarz (2008), as well as Lee et al. (2015), showed that under circumstances that prompt a critical mindset (i.e., low processing fluency) and calling the cooperative context into question (i.e., “fishy” smells), the illusion is reduced. In fact, one might consider that the cooperative communication mind-set feeds into the partial-matching hypothesis. In a cooperative setting, a partial match might suffice, while in a competitive setting people should consider all the presented information. In addition, the conclusion by Reder and Kusbit (1991; Exp. 1) rests on the assumption that people fully remember and comply with the instructions. In other words, the better performance in the gist condition follows because it matches participants’ standard low effort mode, which presumes a cooperative context. However, as stated, manipulating the context to be less cooperative reduces the illusion.

To address the cooperative context explanation more directly, we investigated the Moses illusion in a multiple-choice format (Exps. 1 and 2). If the illusion persists if the correct answer for distorted questions is present on each trial using the multiple-choice format, this would exclude a cooperative communication explanation of the illusion. As the correct response for distorted questions is present on each trial, a cooperative context is directly realized. If the typical Moses illusion is due to a violation of the maxim of cooperation (Grice, 1975), the effect should disappear when participants are repeatedly reminded through the response option “can’t say” that the question itself might be wrong. In addition, choosing one of four answers should be similar effortful for each answer. The exception would be a response option that asks for combinations of other options (e.g., “none of the above”), which is not recommended for multiple-choice tests (see Butler, 2018) and not implemented here.

Beyond the theoretical implications, the multiple-choice format has the practical advantages that it facilitates data collection. In previous experiments, participants wrote down their response or responded verbally. Both cases required additional coding. The multiple-choice format eliminates this step and allows for standardized coding via computer-scripts.

Second, building on Experiments 1 and 2, we investigate the role of motivation by monetary incentives (Exps. 3 and 4). As delineated, in particular the partial matching hypothesis (Kamas et al., 1996; Reder & Cleeremans, 1990) relies on the default of low-effort processing and assumes that participants do not thoroughly process the presented questions. This is in line with the classic “cognitive miser” hypothesis (Kurzban et al., 2013; Zipf, 1949). However, if answers have monetary consequences, participants should be motivated to read and process the questions more carefully (e.g., Terborg & Miller, 1978), thereby reducing the illusion. Monetary incentives are usually a good tool to increase motivation in quantitative tasks (e.g., Cerasoli et al., 2014). In other words, if the Moses illusion depends on a lack of motivation, then incentivizing responses should reduce the illusion. Conversely, if incentives do not influence the illusion, one may conclude that people are unable rather than unmotivated to avoid the illusion.

In the following, we report two experiments that investigate the Moses illusion in a multiple-choice format, and building on these, two further experiments that incentivize participants’ responses in the multiple-choice format. Experiment 1 presents an initial test if the Moses illusion persists with a multiple-choice format. Experiment 2 replicates Experiment 1 and varies procedural aspects of the multiple-choice format. The multiple-choice format realizes a cooperative context, as the correct answer is available on every trial. Experiments 3 and 4 then use this multiple-choice format to investigate the influence of motivation by monetary incentives on the Moses illusion. By incentivizing responses, we aim to increase participants’ motivation to process the questions thoroughly. If the illusion persists nevertheless, we will have evidence that it does not depend on people’s shallow processing of the questions, in particular when the multiple-choice format makes the correct answers readily available.

We report how we determined our sample sizes, all data exclusions (if any), all manipulations, all measures, and all studies we did in this research line so far. In addition, we pre-registered Experiments 2, 3, and 4; these preregistrations, as well as data and materials for all four studies, can be found at: https://osf.io/8dzkt/?view_only = 24b06bb6dd364d66913521dad3c58836.

## Experiment 1: Moses and multiple-choice

Experiment 1 investigated if the Moses illusion persists in a multiple-choice format across a series of distorted (“Moses”) and undistorted (“Noah”) questions. If the illusion follows from violations of cooperative communication, the multiple-choice format should substantially reduce the illusion, as the correct response (“can’t say”) is available on every trial.

### Method

#### Materials

We used 40 questions in two versions each: a distorted version (i.e., “How many animals of each kind did Moses take on the ark?”) and an undistorted version (i.e., “How many animals of each kind did Noah take on the ark?”). We used the 26 questions and their respective answers from Park and Reder (2004) and replaced 14 questions because of apparent cultural knowledge differences between the USA and Germany. We thus generated 14 new questions and answers for a final set of 40 questions. Appendix 1 provides the list of distorted and undistorted questions. We tested people’s knowledge of these 40 questions’ critical term in the student population that serves as the main source of participants for our lab with open-ended questions (i.e., “Which biblical figure took two animals of each kind on the Ark?”). Six coders rated the responses from 120 participants; three coders rated the first half of the questions and three different coders rated the second half. Participants answered the open-ended questions correctly 69.7% of the time.^1^

#### Participants and design

We had no assumptions about the relevant effect size. Based on the recommendations by Ledgerwood (2015), we gathered data of 100 students who participated in exchange for a bar of chocolate and were recruited on campus to participate in a lab-based study. Previous studies typically used between 20 and 40 participants per condition. Two participants did not complete the study, leading to a final sample of 98 (*M*_age_ = 23.10 years, *SD* = 6.28; 43 female, 55 male).

We manipulated within-participants question type (“distorted” vs. “undistorted”); half of the questions appeared as distorted questions and half appeared as undistorted questions. Each question only appeared as either distorted or undistorted. Each question had four different response options. The first response option (e.g., “two”) could be correct or incorrect, depending on the question type (i.e., “How many animals of each kind did Noah take on the Ark?” vs. “How many animals of each kind did Moses take on the Ark?”). The second response option was always incorrect (e.g., “three” to the question “How many animals of each kind did Noah take on the Ark?”). The third response option was “can’t say,” which could be correct or incorrect, depending on the question type (e.g., incorrect in response to the question “How many animals of each kind did Noah take on the Ark?”, but correct in response to the question “How many animals of each kind did Moses take on the Ark?”). The fourth response option was “don’t know,” which counted as neither correct nor incorrect and effectively allowed participants to skip a question for lack of relevant knowledge. The order of response options 3 and 4 was swapped for Experiment 1; for consistency, we report all results using the order that Experiments 2–4 employed.

#### Procedure

The data were collected together with another study that investigated the influence of females wearing a hijab on participants’ responses. After finishing this unrelated study, participants continued with the present experiment within a Qualtrics survey. The survey informed participants that they had to answer 40 multiple-choice trivia questions and that they could skip a question by choosing the “don’t know” response. It also explained that unanswerable questions could appear, in which case the correct response would be “can’t say.” Specifically, the instructions read “It is possible that some questions appear that cannot be answered. In that case, please select the response option ‘Can’t say’. If you don’t know the answer to a question, please select the response option ‘I don’t know’.” The questions appeared individually in a new randomized order for each participant. Participants had to choose one the of the responses to proceed. After answering the 40 questions, participants answered two more questions pertaining to the previous task and indicate if they had suspicions about the aim of the study. Afterwards, the experimenter thanked and debriefed participants.

### Results

Table 1 presents participants’ mean response frequencies to distorted questions (i.e., “How many animals of each kind did Moses take on the ark?”) and to undistorted questions (i.e., “How many animals of each kind did Noah take on the ark?”). As Table 1 shows, participants provided erroneous “Moses” responses almost half the time (*M* = 9.74 out of 20 questions). In addition, participants provided substantially more “can’t say” responses for distorted (*M* = 7.88) compared to undistorted (*M* = 2.12) questions, suggesting that participants understood the task.

**Table 1:** Mean frequencies in Experiment 1 of the four different response options across 40 questions as a function of question type (distorted vs. undistorted)

Question type	Response			
	1	2	3	4
Undistorted	15.17 (3.22)	1.23 (0.97)	2.12 (2.98)	1.48 (1.70)
Distorted	9.74 (4.27)	0.62 (1.11)	7.88 (4.74)	1.77 (1.73)

*Note*. Response 1 represents the “Moses” response for distorted questions and the correct response for undistorted questions. Response 2 represents the false alternative. Response 3 represents the “can’t say” option (correct for distorted questions), and response 4 represents the “don’t know” option (i.e., lack of knowledge for the topic)

Standard deviations are given in parentheses

To provide an inferential statistical test, we coded Moses responses as 1 and all other response types as 0 before adding up all values to compute the total number of Moses responses for each participant. We then compared the number of Moses responses (responses to distorted questions as if the question were undistorted, i.e., “Moses”) to the number expected at chance level of 1/3 (6.67). We chose 1/3 instead of 1/4 because the fourth response option is a “skip” rather than an actual answer and 1/3 is a more conservative comparison. We used a Welch-test to compare the average number of Moses responses to this number and found a significant difference, *M* = 9.73, 95% confidence interval (CI) [8.88, 10.59], *t*(97) = 7.10, *p* < .001, *d* = 0.71.

To check whether the multiple-choice format reduced the illusion, we compared our rate of Moses responses (49%) to the rates reported in Reder and Kusbit (1991). For our comparison, we chose the literal condition, which is identical to our setup. Their Moses response rates were all lower (33% in Experiment 1, 35% in Experiments 2 and 3, 32% in Experiment 4, all taken from the literal task condition), which suggests that our multiple-choice format did not diminish, but even fostered, the illusion. Of course, the present data and the data by Reder and Kusbit differ on many aspects, such as the time of collections, the participant sample, and the stimuli; thus, the numerical difference cannot be attributed to the multiple-choice format, but it is important to note that the multiple-choice format did not produce completely different results.

### Discussion

Experiment 1 showed a Moses illusion with a multiple-choice format. The constant presence of the “can’t say” option should have reminded participants that some of the questions are distorted questions and the effort involved should be similar for all response options. Participants nonetheless selected the Moses response in almost 50% of the distorted cases. Thus, the Moses illusion also appears in a multiple-choice format, which provides strong support that the illusion is not due to participants misunderstanding of a cooperative communication setting.

There are three limitations with regards to conclusions on the Moses illusion in a multiple-choice format, though. First, participants might have learned that the first response option is the relevant one and therefore might have preferentially selected this response option. In other words, because participants always saw the four response options in the same constant order, and the first response is correct in 50% of the cases, they might have inferred that the first response is always the correct response. Response behavior based on such an inference might mimic a Moses illusion in a multiple-choice format. The underlying reason for choosing the “Moses” responses would thus not be a Moses illusion proper, but a learned response bias from the undistorted questions.

Second, we did not check if participants have, in principle, the relevant knowledge. We included the “don’t know” response, but this might not be an accurate representation of what participants know because there are no consequences to guessing blindly.

Third, and finally, participants might have confused the “can’t say” with the “don’t know” options. Although “can’t say” is the response often requested in research on the Moses illusion for distorted items with open formats (e.g., Reder & Kusbit, 1991), it might not be ideally suited for a multiple-choice format.

Experiment 2 addresses these three concerns by comparing a fixed order of response options with a shuffled order, by checking for participants knowledge in the same experiment, and by adjusting the “can’t say” response option to specify its meaning more precisely.

## Experiment 2: Moses and shuffled multiple-choice

Experiment 1 showed that, in principle, a Moses illusion is also apparent in a multiple-choice format. To address the three limitations discussed above, Experiment 2 included three changes. First, to address the problem that Experiment 1 might have introduced a response bias for the first option, Experiment 2 manipulated between participants whether the order of response options was constant or shuffled anew for each question. Second, to address whether the illusion in the multiple-choice format depends on lack of knowledge about the questions being presented, Experiment 2 asked participants direct, open-ended questions at the end about each question’s critical term (e.g., “Which biblical figure took two animals of each kind on the Ark?”). Third, we changed the “can’t say” option to “This question can’t be answered in this form.” Different from Experiment 1, we collected the data online using Amazon’s Mechanical Turk platform.^2^

### Method

#### Materials

We translated our newly created questions to English, but replaced questions culturally specific to Germany with additional questions from Park and Reder (31 questions from Park & Reder, nine of our own questions translated to English). The whole set of questions for Experiment 2 is available in Appendix 2.

#### Participants and design

We again manipulated question type (“distorted” vs. “undistorted”) within participants. We manipulated response option order (“shuffled” vs. “fixed”) between participants. We aimed for the same sample size as Experiment 1. However, as we manipulated the randomized presentation of the options and the fixed presentation between participants, we aimed to collect data from 200 participants in total (100 per cell; see preregistration). We collected data from 205 Amazon Mechanical Turk workers who participated for $2.85. As preregistered, we excluded three participants because they indicated low concentration during the study, and two apparent bots, leaving 200 participants in the final sample for analysis (*M*_age_ = 40.0 years, *SD* = 11.3; 82 female, 115 male, three “prefer not to say”).

#### Procedure

Participants were redirected to the Qualtrics survey from the online platform Amazon Mechanical Turk. The survey randomly assigned participants to one of two conditions. The fixed condition replicated Experiment 1. In the shuffled condition, response options order was randomized anew for each question. Question order was also randomized in both conditions.

In both conditions, participants first read and agreed to an informed consent form and the instructions informed them about the procedure of the experiment and asked them to not look up any answers online. Different from Experiment 1, both conditions used the response option “This question can’t be answered in this form” instead of the option “can’t say.” The instructions stressed the difference between “This question can’t be answered in this form” and “Don’t know” response options and in what cases they should be used. Specifically, the instructions read “Some of these questions are impossible to answer. In that case, the correct response option is: ‘This question can’t be answered in this form.’ If you don't know the answer to a question, please select the response option ‘Don't know’”.

Upon completing the 40 multiple-choice questions, participants responded to 40 corresponding open-ended format questions (e.g., for the typical Moses question, participants responded to “Which biblical figure took two animals of each kind on the Ark?”), checking if participants have the relevant knowledge to answer the multiple-choice questions correctly. Finally, participants provided demographic information and indicated how concentrated they were during the study on a scale from one to six.

### Results

We excluded 192 responses because their respective response times were more than three SDs above the mean for that specific participant (i.e., 2.4% of all responses).^3^ Please note that this also served as our exclusion criterion for potential cheating (i.e., searching for correct answers online), as searching for an answer online should increase response latencies relatively within participants, and if a given participant searches for all answers, relatively to other participants (see also Footnote 3). Table 2 presents the mean frequencies of different response types to the multiple-choice questions.

**Table 2:** Mean frequencies in Experiment 2 of the four different response options across all 40 questions as a function of question type (distorted vs. undistorted) and response order (constant vs. shuffled)

Response order	Question type	Response			
		1	2	3	4
Fixed	Undistorted	17.22 (3.07)	0.73 (2.14)	0.52 (0.87)	1.14 (1.90)
	Distorted	10.89 (4.66)	0.74 (1.90)	6.12 (5.08)	1.63 (2.40)
Shuffled	Undistorted	17.09 (3.02)	0.53 (1.40)	0.40 (0.75)	1.47 (2.02)
	Distorted	10.15 (4.59)	0.72 (1.60)	6.80 (5.78)	1.94 (2.43)

Note. Response 1 represents the “Moses” response (or correct response for undistorted questions), response 2 the false alternative, response 3 represents the “can’t say” option (correct for distorted questions), and response 4 represents the “don’t know” option (i.e., lack of knowledge for the topic. Individual rows do not add up to 20 because of excluded responses. Please not that in the shuffled condition, response number does not indicate question order

Standard deviations are given in parentheses

Two important points are visible from Table 2’s descriptive statistics. First, overall, we replicated the Moses illusion with an online American sample using the multiple-choice format; participants provided erroneous Moses responses more than half the time (52.6%). As in Experiment 1, we compared the average amount of Moses responses to the amount based on the chance level of 1/3 (6.67). A Welch-test showed a significant difference, *M* = 10.52, 95% CI [9.87, 11.16], *t*(199) = 11.75, *p* < .001, *d* = 0.83, between the frequency of Moses responses and chance level.

Second, there is only a small difference between the fixed and the shuffled conditions. The preregistered Welch-test between the constant (*M* = 10.89) and shuffled (*M* = 10.15) conditions was not significant, *t*(197.74) = 1.13, *p* = .259. To provide statistical evidence beyond the null results that the illusion strength is equivalent between conditions, we followed up the Welch-test with an equivalence test (Lakens et al., 2018). Before data collection, we used the TOSTER package in R (Lakens, 2017) to run a power analysis based on our sample size of 100 per cell and an alpha level of α = .05 and 90% power. This resulted in lower and upper equivalence bounds of Δ_*L*_ = -.47 and Δ_*U*_ = .47, so we preregistered an interval containing an effect of |*d*| < .47 to be equivalent. The equivalence test was significant, 90% CI [-.034, 1.82], *t*(197.74) = -2.19, *p* = .015. Based on the equivalence test and the null-hypothesis test combined, we can conclude that the observed effect is statistically not different from zero and statistically equivalent to zero.

In addition, participants provided on average substantially more “This question can’t be answered in this form” responses for distorted (*M* = 0.46) compared to undistorted (*M* = 6.46) questions, suggesting again that participants understood the task.

#### Moses illusion as a function of knowledge

Six independent coders (three for each half of the questions) coded the open-ended answers for correctness (0 = incorrect, 1 = correct). Their interrater reliability was very high (Fleiss’ kappa = .95). If coders disagreed on a response’s correctness, we used the value that the majority of the coders agreed upon. Similar to our student sample, participants knew the critical term for a given question in 73.2% of cases.

To test if knowledge influences illusion strength, we excluded all responses for which the coding indicated lack of knowledge of the topic. First, to test if the Moses illusion persists in this dataset with exclusions based on knowledge of the topic, we again ran a Welch test comparing the percentage of Moses responses against the chance level of 1/3. We used ratios instead of frequencies in this case to account for the different number of exclusions per participant. We found a significant difference, *M* = 0.52, 95% CI [0.48, 0.56], *t*(199) = 9.57, *p* < .001, *d* = 0.68. Second, to test if the exclusions influenced the pattern between conditions, we repeated the Welch test and equivalence test between conditions in the dataset with knowledge exclusions. Again, the frequency of Moses responses did not differ between shuffled (*M* = 6.0) and fixed (*M* = 6.7) conditions, *t*(191.64) = 1.28, *p* = .203, and the equivalence test was significant, 90% CI [-0.19, .1.49], *t*(191.64) = -2.04, *p* = 0.02. Third, we directly compared illusion strength in terms of ratios in the dataset without knowledge exclusions (*M* = 0.54) with illusion strength in the dataset with knowledge exclusions (*M* = 0.52). A paired Welch test showed that the 0.02% reduction was significant, *t*(199) = 2.02, *p* = .044, *d* = .14. However, importantly, even in the dataset with exclusions, participants still gave Moses responses more than half of the time.

### Discussion

Experiment 2 successfully replicated the Moses illusion using a multiple-choice design. Experiment 2 thereby addresses three concerns about Experiment 1. First, the non-significant difference between the fixed and shuffled conditions with considerable power, together with the significant equivalence tests, make an explanation of the illusion in terms of a response bias for the first response unlikely. In addition, also the change from a laboratory to an online setting had no major effects.

Second, the illusion was also strongly present for a strict version of the illusion, in which we only considered responses for which participants provided the correct response in corresponding open-ended questions. Excluding responses for which participants could not recall the critical term led to a significant reduction in illusion strength, but this reduction was very small (i.e., from 0.54 to 0.52, in terms of ratios). Thus, we are confident that we are addressing a true illusion, and not another form of guessing bias. This is also relevant for Experiment 1, where we found a similar percentage of knowledge for the critical terms. It is also important to keep in mind that the open-ended knowledge check for the critical term is the most conservative test, as participants might recognize that it was not Noah who took two animals on the Ark, but they might not be able to produce the correct term “Moses” in a free-recall format.

Third, the more explicit labeling of the correct response to distorted question as “This question can’t be answered in this form” did also not produce strong changes. The illusion strength was highly similar to Experiment 1, given the change in settings.

Together, the results of Experiments 1 and 2 make an explanation of the Moses illusion in terms of Grice’s (1975) maxim of cooperation unlikely. The multiple-choice format presents the correct responses for both distorted and undistorted questions at each trial, fully realizing a cooperative communication setting, that is, the questions have the correct answers available on each trial. The present data thereby substantiate and extend the conclusions by Reder and Kusbit (1991). The experiments also show that a multiple-choice version seems suitable to capture the Moses illusion, which represents a strong practical facilitation of research on this interesting illusion.

Having the feasibility of the multiple-choice format established, we used this format to investigate the role of motivation by monetary incentives on the illusion’s strength.

## Experiment 3: Moses and money

Experiment 3 made responses for participants relevant by providing monetary incentives for each response. If the illusion follows from participants superficial processing of the questions, then incentives should decrease the strength of the illusion. Experiment 3 used Experiment 1’s fixed multiple-choice format. In addition to the within variation of question type (i.e., distorted vs. undistorted), we implemented three between-participants incentive conditions. A “no-incentives” condition replicated Experiment 1 besides the differential compensation (i.e., Experiment 3 offered payment, while Experiment 1 offered a chocolate bar). A “low-incentives” condition awarded 15 cents per correct response and subtracted 15 cents per incorrect response. Given the 40 questions, participants could thus earn up to 6€ in the “low-incentives” condition. A “high-incentives” condition awarded 30 cents per correct response and subtracted 30 cents per incorrect response. Given the 40 questions, participants could thus earn up to 12€ in the “high-incentives” condition. In comparison to Experiment 1, the penalty for guessing should deter participants from responding anything other than “don’t know” if they do not have the relevant knowledge or if they feel unsure, which makes this response option an approximation of a knowledge check.

If the Moses illusion is due to participants’ lack of motivation and the following superficial processing of the questions, we would expect a main effect of condition on erroneous “Moses” responses: Motivation and, subsequently, attention and depth of question processing, should increase with incentives. Thus, we would expect a linear trend, with high incentives leading to more correct responses than low incentives and low incentives leading to more correct responses than no incentives. Based on this reasoning, we pre-registered a linear trend from the no-incentives to the high-incentives condition. We did not specify a significant difference between the high-incentives and the low-incentives conditions. However, there should be no quadratic trend (i.e., less correct responses in the high-incentives condition compared to the low-incentives condition).

### Method

#### Materials

We used the same questions and responses as in Experiment 1. We adjusted one question due to an ambiguity in the question. Originally, we asked “What is the name of the prize awarded in Sweden for significant contributions in the fields of science *and peace*?” (undistorted). We changed this to “What is the name of the prize awarded in Sweden for significant contributions in the field of science?” (undistorted) because the Nobel Peace Prize is awarded in Oslo, Norway.^4^

#### Participants and design

Based on the sample size of Experiment 1 (n = 100), we pre-registered a sequential analysis based on Lakens (2014) to reduce the cost of the experiment. We pre-registered to gather data of 150 participants (50 per condition) and then stop collecting data if we find the predicted linear trend from the no-incentives condition to the high-incentives condition. If this was not the case, we planned to collect data for the full 100 participants per condition with an adjusted *p*-value of *p* < .0294 (see Lakens, 2014, p. 703). At 150 participants, the pre-registered analyses showed no effect and so we continued data collection with the goal of 300 participants. Ultimately, 318 students participated for base payment of 4€ plus the incentives in the incentivized conditions. We excluded three participants who indicated concentration of less than 3 on a scale of 1–6 during the experiment, leading to the final sample of 315 participants (*M*_age_ = 23.17 years, *SD* = 5.87; 187 female, 123 male, two other, three “prefer not to say”). We recruited all participants on a university campus for participation in a lab-based study.

The computer program randomly assigned participants to one of three incentives conditions. The total amount could not go below zero and participants received their final score in cents in addition to a flat payment of 4€. Again, participants could earn up to 12 € in the high-incentives condition and up to 6 € in the low incentives condition in addition to their flat payment. Participants in the no incentives condition did not gain or lose money during the experiment. All participants expected to receive 4€ during recruitment, before we randomly assigned them to conditions. In addition to collecting responses to the questions, the program also collected response times for each question from showing the question and the participant clicking the “next” button. The question and response presentation were identical to Experiment 1.

#### Procedure

Experimenters welcomed participants in the lab and seated them in a cubicle in front of a computer and launched a Python program that led participants through the rest of the experiment. Participants read and agreed to an informed consent form and the program informed them about the procedure of the experiment. It asked them to turn off their smartphones to deter cheating and explained the incentive system. The response options were similar to Experiment 1. The instructions specifically mentioned the “can’t say” and “don’t know” response options and explained that the former was the correct response to non-answerable questions while the latter did not affect the point total and could be used to skip a question. In comparison to Experiment 1, this should help avoid confusion in regards to meaning of the response options. After the 40 questions, participants answered demographic questions and how concentrated they were during the study before the experimenter thanked, debriefed, and paid them.

### Results

Table 3 shows the percentage of different response types to distorted questions and undistorted questions as a function of no, low, and high incentives. Overall, the results replicated Experiment 1. As the table shows, across conditions, participants showed a substantial Moses illusion. They provided on average Moses responses for 8.85 out of 20 questions.

**Table 3:** Mean frequencies in Experiment 3 of the four different response options across all 40 questions as a function of question type (distorted vs. undistorted) and incentives (high vs. low vs. none)

Incentives	Question type	Response			
		1	2	3	4
High	Undistorted	15.64 (3.16)	0.16 (0.40)	2.22 (2.11)	1.97 (2.24)
	Distorted	8.05 (4.80)	0.78 (1.00)	8.93 (5.31)	2.24 (2.12)
Low	Undistorted	15.85 (2.85)	0.38 (0.75)	2.19 (1.98)	1.58 (1.84)
	Distorted	8.93 (4.43)	0.58 (0.82)	8.61 (4.95)	1.88 (1.95)
None	Undistorted	15.65 (3.05)	0.49 (1.02)	2.15 (2.47)	1.7 (1.66)
	Distorted	9.58 (4.30)	0.61 (1.19)	7.95 (4.98)	1.87 (1.96)

*Note*. Response 1 represents the “Moses” response for distorted questions and the correct response for undistorted questions. Response 2 represents the false alternative. Response 3 represents the “can’t say” option (correct for distorted questions), and response 4 represents the “don’t know” option (i.e., lack of knowledge for the topic)

Standard deviations are given in parentheses

#### Confirmatory analyses of incentive effects

We computed illusion strength identically to Experiment 1. We then checked whether the basic Moses effect persisted by comparing the mean number of Moses responses to the number based on the chance level of 1/3 (6.67). A Welch-test showed a significant difference, *M* = 8.85, *t*(314) = 8.53, *p <* .001, *d* = 0.48, between the frequency of Moses responses and chance level.

To analyze the mean number of Moses responses as a function of incentives, we submitted these data to a one-way ANOVA with condition (incentives: high vs. low vs. no) as the sole between-participants factor. There was no significant main effect for condition, *F*(2,312) = 3.01, *p* = .051, η_p_^2^ = .019. However, as pre-registered, the linear contrast from high to no incentives was significant, *t*(312) = 2.44, *p =* .015, *d* = 0.28. The quadratic trend was not significant, *t*(312) = -0.23, *p* = .820, *d* = -0.03.

To analyze the mean number of correct responses, we coded correct responses as 1 and all other response types as 0 before adding up all values to compute the mean number of correct responses. We pre-registered a main effect of condition and a linear trend from the no-incentives condition to the high-incentives condition with participants in the high-incentives condition giving the most correct responses. The data was submitted to a one-way ANOVA with incentives (high vs. low vs. none) as the between factor. Contrary to our pre-registered hypotheses, the main effect for condition on correct responses was not significant, *F*(2,312) = 1.10, *p* = .335, *η*_p_^2^ = .007; and neither the linear trend, *t*(312) = -1.36, *p* = .176, *d* = -0.15, nor the quadratic trend were significant, *t*(312) = -0.60, *p* = .551, *d* = -0.07.

#### Exploratory analyses

As an exploratory analysis, we also compared the average proportion of type 4 responses (skips) between conditions. From the distribution of response types, it seems that participants skipped questions more often in the high-incentives condition compared to the other two. This would make sense, because the high-incentive condition also has the highest losses for incorrect questions. However, the main effect for condition was not significant, *F*(2,312) = 1.45, *p* = .236, η_p_^2^ = .009, and neither the linear trend, *t*(312) = -1.34, *p* = .180, *d* = -0.15, nor the quadratic trend were significant, *t*(312) = 1.05, p = .295, *d* = 0.12.

The supplements also provide and exploratory analyses of the response times.

### Discussion

We again replicated the basic Moses effect using multiple-choice questions. In addition, incentives did influence the frequency of Moses responses. We found the predicted linear trend from high to no incentives; participants in the high-incentives condition provided less Moses responses compared to the no incentives condition. However, the effect was much weaker than anticipated. In fact, a potential additional payment of 12€ (around $14) reduced the illusion only by 16% and necessitated 300 participants to show it statistically (see sequential analysis).

Based on feedback from our lab meetings, one reason could be again the wording of the different response options. Specifically, as we used the response option from Experiment 1, participants may construe the phrase “Can’t say” as “I can’t answer this” which would be very close in meaning to “I don’t know.” Even though we made sure to explain the different response options in the instructions, this could nevertheless have influenced participants’ responses in the incentivized version, diminishing the potential incentive influence. We thus aimed to replicate the surprising result from Experiment 3 (i.e., the small incentive effect) with the changed response option from Experiment 2.

## Experiment 4: Moses and money replicated?

We aimed to replicate the basic Moses effect and the linear influence of incentives on the illusion’s strength.

### Method

#### Materials, participants, design, and procedure

We changed the response option “Can’t say” to “This question can’t be answered in this form,” which is also the response option used in Experiment 2. Otherwise, the materials were identical to the ones used in Experiment 3. As for Experiment 3, we pre-registered a sample of 300 participants. 298 students participated for payment (*M*_age_ = 22.19 years, *SD* = 4.78; 146 female, 148 male, four other) and were again recruited on a university campus. The design and procedures were identical to Experiment 3.

### Results

Table 4 shows the percentage of different response types to distorted questions and undistorted questions as a function of no, low, and high incentives. Across conditions, participants again showed a Moses illusion in a multiple-choice format with incentives. Out of 20 distorted questions, they on average provided Moses responses for 7.74 questions. This is an approximate average 5% drop (or one question) in comparison to Experiment 3, which could be a direct result of the change of phrasing for the “can’t say” option.

**Table 4:** Mean frequencies in Experiment 4 of the four different response options across all 40 questions as a function of question type (distorted vs. undistorted) and incentives (high vs. low vs. none)

Incentives	Question type	Response			
		1	2	3	4
High	Undistorted	15.31 (2.87)	0.35 (1.1)	2.18 (1.92)	2.15 (2.11)
	Distorted	7.49 (4.25)	0.66 (1.05)	9.33 (5.02)	2.52 (2.34)
Low	Undistorted	15.19 (2.96)	0.33 (0.73)	2.21 (2)	2.26 (2.51)
	Distorted	7.49 (4.17)	0.46 (0.76)	9.33 (4.95)	2.71 (2.84)
None	Undistorted	15.76 (2.87)	0.41 (0.74)	2.29 (2.47)	1.54 (1.67)
	Distorted	8.24 (4.07)	0.54 (0.85)	9.58 (4.92)	1.64 (1.76)

*Note*. Response 1 represents the “Moses” response for distorted questions and the correct response for undistorted questions. Response 2 represents the false alternative. Response 3 represents the “can’t say” option (correct for distorted questions), and response 4 represents the “don’t know” option (i.e., lack of knowledge for the topic)

Standard deviations are given in parentheses

#### Confirmatory analyses of incentive effects

A Welch-test between average number of Moses responses per participant and the number expected from chance level (6.67) was again significant, *M* = 7.77, *t*(297) = 4.46, *p* < .001, *d* = 0.26.

We computed illusion strength as in Experiment 3. We submitted these data to a one-way ANOVA with incentive condition (high vs. low vs. no) as the between factor. There was no incentive condition main effect, *F*(2,295) = 1.07, *p* = .346, η_p_^2^ = .007, and different from Experiment 3, the linear contrast between high, low, and no incentives was also not significant, *t*(295) = 1.26, *p* = .208, *d* = 0.15; neither was the quadratic trend, *t*(295) = 0.73, *p* = .467, *d* = 0.08. Thus, while the effect is numerically in the expected direction from the high (*M* = 7.49) to the no incentives (*M* = 8.24) condition, we did not replicate the influence of incentives on the strength of the Moses illusion.

To analyze the mean number of correct responses, we coded correct responses as 1 and all other response types as 0 before adding up all values to compute the mean number of correct responses. We submitted this data to a one-way ANOVA with incentives (high vs. low vs. none) as the between factor. As in Experiment 3, contrary to our predictions, the incentives main effect was not significant, *F*(2,295) = 0.62, *p* = .538, η_p_^2^ = .004. The linear trend was also not significant, *t*(295) = 0.88, *p* = .380, *d* = 0.10, and the quadratic trend was also not significant, *t*(295) = 0.68, *p* = .495, *d* = 0.08.

#### Exploratory analyses

As an exploratory analysis identical to that of Experiment 3, we also compared the average proportion of type 4 answers (skips) between conditions. From the distribution of response types, it seems that participants skipped questions more often in the high-incentives condition compared to the other two. This would make sense since the high-incentive condition also has the highest losses for incorrect questions. This time, the main effect for condition was significant, *F*(2,295) = 5.69, *p* = .004, *η*_p_^2^ = .037, and the linear trend was also significant, *t*(295) = 2.62, *p* = .009, *d* = -0.30. The analysis of the response times is presented in the supplement.

### Discussion

While we replicated the overall Moses effect, we did not replicate the influence of incentives on the illusion; different from Experiment 3, the linear trend between the three incentives conditions was not significant. Also different from Experiment 3, the main effect of incentives on skips was significant and the negative linear trend indicates that participants skipped more questions as incentives increased.

Overall, the average number of correct responses was unaffected by incentives, but the significance pattern regarding illusion strength as a function of incentives was inconsistent between Experiments 3 and 4. While the incentives linearly decreased the frequency of Moses responses in Experiment 3, we found this trend only numerically, not statistically, in Experiment 4. Because of the different result patterns, we analyze the data from Experiments 3 and 4 together. As the difference between significant (Experiment 3) and non-significant (Experiment 4) is by itself not necessarily significant, this analysis allows us to test this significance difference of the linear trend by an interaction via Experiment.

### Combining Experiments 3 and 4

Table 5 shows the frequency of different response types to distorted questions and undistorted questions separated by conditions. We added experiment as a factor to the previous ANOVA for a 3 (incentives: high vs. low vs. no) x 2 (Experiment: 3 vs. 4) ANOVA with percentage of Moses response as dependent variable. This ANOVA showed a significant incentive main effect, *F*(2, 607) = 3.54, *p* = .030, *η*_p_^2^ = .012. This incentive main effect was due to the expected the linear trend from high to no incentives, *t*(607) = 2.64, *p =* .009, *d* = 0.21.The quadratic trend was not significant, *t*(607) = 0.34, *p* = .736, *d* = 0.03. Importantly, the linear trend did not interact with Experiment (3 vs. 4), *F*(1, 607) = 0.82, *p* = .366. However, there was an Experiment main effect, *F*(1, 607) = 9.88, *p* = .002, *η*_p_^2^ = .016. Participants in Experiment 3 provided more Moses responses (*M* = 8.85, *SD* = 4.54) than participants in Experiment 4 (*M* = 7.77, *SD* = 4.16).

**Table 5:** Combined mean frequencies in Experiments 3 and 4 of the four different response options across all 40 questions as a function of question type (distorted vs. undistorted) and incentives (high vs. low vs. none)

Incentives	Question type	Response number			
		1	2	3	4
High	Undistorted	15.48 (3.02)	0.26 (0.82)	2.2 (2.01)	2.06 (2.17)
	Distorted	7.78 (4.54)	0.72 (1.03)	9.13 (5.16)	2.37 (2.23)
Low	Undistorted	15.53 (2.92)	0.36 (0.74)	2.2 (1.99)	1.91 (2.21)
	Distorted	8.24 (4.35)	0.52 (0.79)	8.96 (4.95)	2.28 (2.45)
None	Undistorted	15.71 (2.95)	0.45 (0.89)	2.22 (2.46)	1.62 (1.66)
	Distorted	8.92 (4.23)	0.57 (1.04)	8.75 (5.01)	1.75 (1.86)

*Note*. Response 1 represents the “Moses” response for distorted questions and the correct response for undistorted questions. Response 2 represents the false alternative. Response 3 represents the “can’t say” option (correct for distorted questions), and response 4 represents the “don’t know” option (i.e., lack of knowledge for the topic)

Standard deviations are given in parentheses

We also analyzed the correct responses with a 3 (incentives: high vs. low vs. no) x 2 (Experiment: 3 vs. 4) ANOVA. The main effect for incentives was not significant, *F*(2, 607) = 0.04, *p* = .961, η_p_^2^ = .00, and neither were the main effect for experiment, *F*(1, 607) = 2.07, *p* = .151, *η*_p_^2^ = .00, nor the interaction, *F*(2, 607) = 1.64, *p* = .195, *η*_p_^2^ = .00.

We used the same ANOVA for skips (“don’t know”) as dependent variable. This analysis shows a significant effect of incentive condition, *F*(2, 607) = 4.60, p = .010, *η*_p_^2^ = .01, with a significant linear trend from the high to the no incentives condition, *t*(607) = -2.88, *p* = .004, *d* = -0.23. The quadratic trend was not significant, *t*(607) = -0.96, *p* = .337, *d* = -0.08, and the linear trend did not interact with Experiment, *F*(1, 607) = 1.43, *p* = .232.

### Combined discussion

The pooled data from Experiment 3 and 4 confirms the pattern from Experiment 3: A significant main effect of incentives on the average proportion of Moses responses with a linear trend from the high-incentives condition to the no incentives condition. Combined with the reversed linear trend of skips and the non-significant effect of condition on correct responses, this pattern provides some insights into the underlying mechanisms. While participants gave fewer Moses responses in the high-incentives conditions, their overall number of correct responses did not change. This means that participants in the incentive conditions were more careful and chose the skip option more often. This is probably due to our incentivization system, which involved losing points for incorrect responses but not for skips. However, to be sure, the reduction was small and the reduction effect required high statistical power.

## General discussion

The present research had two goals: We wanted to establish a multiple-choice response format for the Moses illusion and thus rule out the possibility that the illusion is due to a cooperative communication setting, and investigate the effect of motivation on the illusion by monetary incentives for correct responses.

The most important result for the multiple-choice format is that it does not eliminate the illusion. If the illusion were due to participants behaving as cooperative communication partners, understanding the distorted questions correctly and then choosing to respond to it as if it were undistorted, then presenting “can’t say” as a response option should have reduced the illusion. The correct response was available on every trial, which should also remind participants that distorted questions exist and the correct response for those questions is “can’t say.” We found a substantial Moses illusion, ruling out the cooperative communication setting explanation (Grice, 1975) and validating the multiple-choice format.

In addition, the multiple-choice format facilitates research on the illusion, as coding participants’ responses is no longer necessary, which greatly reduces the resources required for studies on the Moses illusion.

A small caveat with regards to the multiple-choice format is that there is research showing that participants tend to avoid a “none of the above” (NOTA) option in multiple choice questions (Blendermann et al., 2020). If one considers the present “can’t say” option as a NOTA variant, this avoidance tendency might contribute to the present illusion. However, given the illusion strength present in the data, it seems unlikely that this tendency is fully responsible for the present effects.

Our motivation manipulation by monetary incentives had the hypothesized effect on the Moses illusion. We expected to find that with enough motivation due to monetary incentives, participants would pay more attention and detect distortions more often. Participants in the high-incentives condition should provide the least Moses responses and participants in the no incentives condition should provide the most Moses responses. However, the incentives effect on the average number of Moses responses was much smaller and less reliable than we expected. While the pooled data of Experiments 3 and 4 provide confidence in the statistical significance of the incentives effect, the practical significance is negligible. We paid participants about 4€ (about $4.50) on average for them to give one less Moses response.

When looking at the increased skips in the incentivized conditions for the combined data, one could argue that the improvement in correct answers (and thus, payment) is not due to increased sensitivity to the Moses illusion, but rather a general response bias to be more careful out of fear of losing money. This result appears similar to the bias shift observed by Kamas et al. (1996), but in their studies, participants’ bias shifted towards “can’t say” responses to any question regardless of distortion. This is an incorrect response for half of the questions, whereas the shift towards skip responses in the present research is not an incorrect response but rather a decision to avoid risk.

Taken together, the data from a total of 914 participants show that there is a strong Moses illusion, even when motivation is high and communication context effects are accounted for. Motivating participants with monetary incentives had some effect, but it does not account for a large part of the variance.

Thereby, our results are best compatible with the partial matching hypothesis, with the qualification that the driving force underlying partial matching is not people’s tendency to avoid effort. Our high-incentives condition should have been enough motivation for a student sample to invest enough effort to detect the distortion. Rather, one must consider a decision threshold model in which the partially matching information in the question seems to suffice to pass the threshold to elicit the wrong “Moses” responses (Reder & Cleeremans, 1990, p. 250). Thus, the Moses illusion may emerge not because people do not pay attention or because they aim to be cooperative communication partners, but because the cognitive system is sufficiently prompted by the question’s content to respond “two” when asked how many animals Moses took on the Ark, even when the stakes are relatively high.

## Conclusion

The Moses illusion is a robust phenomenon that we also observed in a multiple-choice format. This implies that the illusion does not follow from respondents’ attempts to be cooperative communication partners. The multiple-choice context clearly communicates on every trial that questions might be wrong. The multiple-choice format also opens many new venues for research on this intriguing illusion. The motive to avoid effort seems to play a minor role in the emergence of the illusion, as monetary incentives had a significant but numerically small effect. This in turn supports explanations of the Moses illusion that rely on cognitive rather than motivational features.

## Appendix 1

Undistorted questions:

R1

Which type of cigarettes was German chancellor Helmut Schmidt known for?

Menthol cigarettes

Filterless cigarettes

R2

Which resource did the USA suspend troops to Iraq for?

Oil

Solar energy

R3

In which movie does Arnold Schwarzenegger travel back in time to save Sarah Connor?

Terminator 2

Rocky 2

R4

With which instrument did Louis Armstrong become famous?

Trumpet

Violin

R5

Which object does Julie Andrews use to fly at the beginning of the movie “Mary Poppins”?

Umbrella

Broom

R6

Gorbachev was the leader of which communist country?

USSR

USA

R7

Margaret Thatcher was the prime minister of which country?

United Kingdom

France

R8

What year did Germany lose World War II?

1945

1918

R9

Which kind of meat is in the Whopper from Burger King?

Beef

Chicken

R10

What color is Dogmatix’s fur, the dog of Asterix and Obelix?

Black and white

Gray and brown

R11

Which season do we associate with the start of football season, the beginning of school and the trees’ leaves turning brown?

Autumn

Winter

R12

Which statue, given to the USA by France, symbolizes freedom for arriving immigrants at New York Harbor?

Statue of Liberty

Christ the Redeemer

R13

Which part of his body did artist Van Gogh allegedly cut off?

Ear

Nose

R14

What musician won multiple Grammys for their Album „Thriller“?

Michael Jackson

Elton John

R15

What follows „To be or not to be” in Hamlet’s famous soliloquy?

„That is the question.“

„Who knows?“

R16

Who is the video game character and Italian plumber who is Nintendo’s mascot?

Mario

Sonic

R17

Which country is known for cuckoo clocks, chocolate and pocket knives?

Switzerland

Italy

R18

Which political position did Adolf Hitler gain under President Paul von Hindenburg?

Chancellor of the Reich

Mayor

R19

Who found the Glass Slipper lost by Cinderella?

The Prince

The Stepmother

R20

What is the name of the kimono-clad courtesans who entertain Japanese men?

Geisha

Samurai

R21

What is the name of Leonardo da Vinci’s famous painting of a woman that is displayed in the Louvre in Paris?

Mona Lisa

The Scream

R22

What is the name of the device that tells time by measuring the incidence of sunlight on a dial?

Sundial

Oscillator

R23

Who is the cartoon character known for eating spinach to get stronger?

Popeye

Mickey Mouse

R24

What is the name of the comic about Charlie Brown and his dog Snoopy?

Peanuts

Cashews

R25

Who is the dictator of North Korea?

Kim Jong-Un

Fidel Castro

R26

What is the name of the molten rock that travels down mountains after an eruption?

Lava

Mud

R27

Who is the Roman god of war after whom a famous candy bar is named?

Mars

Snickers

R28

Who is the white-bearded man in a red suit who distributes Christmas presents out of his sleigh?

Santa Claus

Rumpelstiltskin

R29

What is the name of the Mexican dip made from avocados?

Guacamole

Salsa

R30

What is the protagonist’s name in Goethe’s “Faust, Part One”?

Dr. Heinrich Faust

Romeo

R31

What is the name of the prize awarded in Sweden for significant contributions in the field of science?

Nobel prize

Academy Award

R32

What is the name of the island located in the south of Italy close to the “boot’s toe”?

Sicily

Island of Elba

R33

What is the name of the TV show about a young Viking boy who always rubs his nose when trying to figure something out?

Wickie and the strong men (English: Vicky the Viking)

Pedia and the smart men

R34

Who is the architect of the famous Eiffel Tower in Paris?

Gustav Eiffel

Oscar Niemeyer

R35

What was the name of the wall in East-Germany that was torn down in 1989?

Berlin Wall

Great Wall of China

R36

How long did Sleeping Beauty fall asleep for, after poking her finger on a spindle?

100 years

2 days

R37

What is the name of the New Year festival celebrated on the 31^st^ of December?

New Year’s Eve

Carnival

R38

How many times did the German football team win the World Cup?

Four times

Never

R39

How many doors does an Advent calendar have?

24

365

R40

How many animals of each kind did Noah take on the Ark?

Two

Three

Distorted questions:

F1

Which type of cigarettes was German chancellor Helmut Kohl known for?

Menthol cigarettes

Filterless cigarettes

F2

Which resource did the USA suspend troops to Iran for?

Oil

Solar energy

F3

In which movie does Sylvester Stallone travel back in time to save Sarah Connor?

Terminator 2

Rocky 2

F4

With which instrument did Lance Armstrong become famous?

Trumpet

Violin

F5

Which object does Audrey Hepburn use to fly at the beginning of the movie “Mary Poppins”?

Umbrella

Broom

F6

Gorbachev was the leader of which capitalist country?

USSR

USA

F7

Margaret Thatcher was the president of which country?

United Kingdom

France

F8

What year did Germany win World War II?

1945

1918

F9

Which kind of meat is in the Whopper from McDonalds?

Beef

Chicken

F10

What color is Getafix’s fur, the dog of Asterix and Obelix?

Black and white

Gray and brown

F11

Which season do we associate with the start of football season, the beginning of school and the trees’ leaves turning green?

Autumn

Winter

F12

Which statue, given to the USA by England, symbolizes freedom for arriving immigrants at New York Harbor?

Statue of Liberty

Christ the Redeemer

F13

Which part of his body did artist Gaugin allegedly cut off?

Ear

Nose

F14

What musician won multiple Emmys for their Album „Thriller“?

Michael Jackson

Elton John

F15

What follows „To be or not to be” in Macbeth’s famous soliloquy?

„That is the question.“

„Who knows?“

F16

Who is the video game character and Italian plumber who is Sony‘s mascot?

Mario

Sonic

F17

Which country is known for cuckoo clocks, gummy bears, banks, and pocket knives?

Switzerland

Italy

F18

Which political position did Adolf Hitler gain under President Otto von Bismarck?

Chancellor of the Reich

Mayor

F19

Who found the Glass Slipper lost by Snow White?

The Prince

The Stepmother

F20

What is the name of the kimono-clad courtesans who entertain Chinese men?

Geisha

Samurai

F21

What is the name of Leonardo da Vinci’s famous painting of a woman that is displayed in the Pompidou in Paris?

Mona Lisa

The Scream

F22

What is the name of the device that tells the temperature by measuring the incidence of sunlight on a dial?

Sundial

Oscillator

F23

Who is the cartoon character known for eating spinach to get smarter?

Popeye

Mickey Mouse

F24

What is the name of the comic about Charlie Brown and his dog Oldie?

Peanuts

Cashews

F25

Who is the dictator of South Korea?

Kim Jong-Un

Fidel Castro

F26

What is the name of the molten rock that travels down mountains after an earthquake?

Lava

Mud

F27

Who is the Greek god of war after whom a famous candy bar is named?

Mars

Snickers

F28

Who is the white-bearded man in a red suit who distributes birthday presents out of his sleigh?

Santa Claus

Rumpelstiltskin

F29

What is the name of the Mexican dip made from artichokes?

Guacamole

Salsa

F30

What is the protagonist’s name in Schiller’s “Faust, Part One”?

Dr. Heinrich Faust

Romeo

F31

What is the name of the prize awarded in Denmark for significant contributions in the field of science?

Nobel prize

Academy Award

F32

What is the name of the island located in the north of Italy close to the “boot’s toe”?

Sicily

Island of Elba

F33

What is the name of the TV show about a young Viking boy who always rubs his ear when trying to figure something out?

Wickie and the strong men (English: Vicky the Viking)

Pedia and the smart men

F34

Who is the architect of the famous Eiffel Tower in Marseille?

Gustav Eiffel

Oscar Niemeyer

F35

What was the name of the wall in West-Germany that was torn down in 1989?

Berlin Wall

Great Wall of China

F36

How long did Rapunzel fall asleep for, after poking her finger on a spindle?

100 years

2 days

F37

What is the name of the New Year festival celebrated on the 31^st^ of January?

New Year’s Eve

Carnival

F38

How many times did Bayern München win the World Cup?

Four times

Never

F39

How many doors does an Advent wreath have?

24

365

F40

How many animals of each kind did Moses take on the Ark?

Two

Three

## Appendix 2

Undistorted questions:

1. What kind of tree did Washington chop down?

Cherry

Palm

2. For what valuable energy resource did the U.S. commit many troops to fight against Iraq?

Oil

Solar Energy

3. In what movie did Arnold Schwarzenegger go back in time to protect Sarah Connor?

Terminator 2

Rocky 2

4. With which instrument did Louis Armstrong become famous?

Trumpet

Violin

5. In the beginning of the movie "Mary Poppins", Julie Andrews floats down from the sky with the aid of what object?

Umbrella

Broom

6. Gorbachev was the leader of what communist country?

USSR

USA

7. What country was Margaret Thatcher prime minister of?

United Kingdom

France

8. What year did Germany lose World War II?

1945

1918

9. What kind of meat is in the Burger King sandwich known as the Whopper?

Beef

Chicken

10. By flying a kite, what did Franklin discover?

Electricity

Gravity

11. What season do we associate with football games, starting school, and leaves turning

brown?

Fall

Winter

12. What statue given to the U.S. by France symbolizes freedom to immigrants arriving in New

York Harbor?

Statue of Liberty

Christ the Redeemer

13. Which portion of his body did the famous artist, Van Gogh, supposedly cut off?

Ear

Nose

14. Who won numerous Grammy awards for his breakthrough album "Thriller"?

Michael Jackson

Elton John

15. What phrase followed "To be or not to be" in Hamlet's famous soliloquy?

“That is the question.”

“Who knows?”

16. Who is the video game character and Italian plumber who is Nintendo’s mascot?

Mario

Sonic

17. What country is famous for cuckoo clocks, chocolate, banks and pocketknives?

Switzerland

Italy

18. What did Goldie-Locks eat at the Three Bears' house?

Porridge

Corn Flakes

19. Who found the glass slipper left at the ball by Cinderella?

The prince

The stepmother

20. What is the name of the kimono-clad courtesans who entertain Japanese men?

Geisha

Samurai

21. What is the name of Leonardo da Vinci’s famous painting of a woman that is displayed in the Louvre in Paris?

Mona Lisa

The Scream

22. What is the name of the instrument that by measuring the angle of the sun's shadow on a

calibrated dial, indicates the time?

Sundial

Oscillator

23. What is the name of the comic strip character who eats spinach to improve his strength?

Popeye

Mickey Mouse

24. Snoopy is a dog in what famous comic strip?

Peanuts

Cashews

25. Who is the dictator of North Korea?

Kim Jong-Un

Fidel Castro

26. What is the name of the molten rock that runs down the side of a volcano during an eruption?

Lava

Mud

27. Who is the Roman god of war that has the same name as a famous candy bar?

Mars

Snickers

28. What is the name of the man in the red suit and long white beard who gives out

Christmas presents from his sleigh?

Santa Claus

Rumpelstiltskin

29. What is the name of the Mexican dip made with mashed-up avocados?

Guacamole

Salsa

30. What is the name of the hit in baseball that allows the batter to run around all the bases and get a run?

Homerun

Touchdown

31. What is the name of the famous prize issued by Sweden for contributions to science and

peace?

Nobel Prize

Academy Award

32. Who began an address with "Four score and seven years ago"?

Abraham Lincoln

John F. Kennedy

33. What is the name of the carved pumpkin displayed on Halloween?

Jack-o’-lantern

Soul cake

34. Who is the architect of the famous Eiffel Tower in Paris?

Gustave Eiffel

Oscar Niemeyer

35. When did the Japanese attack Pearl Harbor?

December 7^th^, 1941

December 7^th^, 1951

36. How long did Sleeping Beauty fall asleep for, after poking her finger on a spindle?

100 years

2 days

37. What is the name of the New Year festival celebrated on the 31st of December?

New Year’s Eve

Carnival

38. What is the name of the man who rode horseback in 1775 to warn that the British were coming?

Paul Revere

Thomas Jefferson

39. In the biblical story, what was Jonah swallowed by?

Whale

Dolphin

40. How many animals of each kind did Noah take on the Ark?

Two

Three

Distorted questions:

1. What kind of tree did Lincoln chop down?

Cherry

Palm

2. For what valuable energy resource did the U.S. commit many troops to fight against Iran?

Oil

Solar Energy

3. In what movie did Sylvester Stallone go back in time to protect Sarah Connor?

Terminator 2

Rocky 2

4. With which instrument did Lance Armstrong become famous?

Trumpet

Violin

5. In the beginning of the movie "Mary Poppins", Audrey Hepburn floats down from the

sky with the aid of what object?

Umbrella

Broom

6. Gorbachev was the leader of what capitalist country?

USSR

USA

7. What country was Margaret Thatcher president of?

United Kingdom

France

8. What year did Germany win World War II?

1945

1918

9. What kind of meat is in the McDonald's sandwich known as the Whopper?

Beef

Chicken

10. By flying a kite, what did Edison discover?

Electricity

Gravity

11. What season do we associate with football games, starting school, and leaves turning

green?

Fall

Winter

12. What statue given to the U.S. by England symbolizes freedom to immigrants arriving in New

York Harbor?

Statue of Liberty

Christ the Redeemer

13. Which portion of his body did the famous artist, Gauguin, supposedly cut off?

Ear

Nose

14. Who won numerous Emmy awards for his breakthrough album "Thriller"?

Michael Jackson

Elton John

15. What phrase followed "To be or not to be" in Macbeth's famous soliloquy?

“That is the question.”

“Who knows?”

16. Who is the video game character and Italian plumber who is Sony‘s mascot?

Mario

Sonic

17. What country is famous for cuckoo clocks, chocolate, stock markets and pocketknives?

Switzerland

Italy

18. What did Goldie-Locks eat at the Three Little Pigs' house?

Porridge

Corn Flakes

19. Who found the glass slipper left at the ball by Snow White?

The prince

The stepmother

20. What is the name of the kimono-clad courtesans who entertain Chinese men?

Geisha

Samurai

21. What is the name of Leonardo da Vinci’s famous painting of a woman that is displayed in the Pompidou in Paris?

Mona Lisa

The Scream

22. What is the name of the instrument that by measuring the angle of the sun's shadow on a

calibrated dial, indicates the temperature?

Sundial

Oscillator

23. What is the name of the comic strip character who eats spinach to improve his sight?

Popeye

Mickey Mouse

24. Snoopy is a cat in what famous comic strip?

Peanuts

Cashews

25. Who is the dictator of South Korea?

Kim Jong-Un

Fidel Castro

26. What is the name of the molten rock that runs down the side of a volcano during an earthquake?

Lava

Mud

27. Who is the Greek god of war that has the same name as a famous candy bar?

Mars

Snickers

28. What is the name of the man in the red suit and long white beard who gives out birthday

presents from his sleigh?

Santa Claus

Rumpelstiltskin

29. What is the name of the Mexican dip made with mashed-up artichokes?

Guacamole

Salsa

30. What is the name of the hit in baseball that allows the batter to run around all the bases and get an out?

Homerun

Touchdown

31. What is the name of the famous prize issued by Denmark for contributions to science and

peace?

Nobel Prize

Academy Award

32. Who began an address with "Four score and twenty years ago"?

Abraham Lincoln

John F. Kennedy

33. What is the name of the carved pumpkin displayed on Thanksgiving?

Jack-o’-lantern

Soul cake

34. Who is the architect of the famous Eiffel Tower in Marseille?

Gustave Eiffel

Oscar Niemeyer

35. When did the Germans attack Pearl Harbor?

December 7^th^, 1941

December 7^th^, 1951

36. How long did Rapunzel fall asleep for, after poking her finger on a spindle?

100 years

2 days

37. What is the name of the New Year festival celebrated on the 31st of January?

New Year’s Eve

Carnival

38. What is the name of the man who rode horseback in 1775 to warn that the French were coming?

Paul Revere

Thomas Jefferson

39. In the biblical story, what was Joshua swallowed by?

Whale

Dolphin

40. How many animals of each kind did Moses take on the Ark?

Two

Three

## Footnotes

1 This test was conducted to address a reviewer’s concerns about the degree to which the questions were common knowledge, which is why it was conducted after Experiment 1 and on a different sample. Experiment 2 addresses the role of knowledge directly.

2 Experiment 2 was factually the last of the four experiments in this research project. It was conducted to address a reviewer’s concern about the fixed order in the response format. In addition, we needed to conduct it online, as the CoViD-19 Pandemic prevented laboratory-based data collection at this time and we could not use an incentivized online version, as participants might easily and readily look up the correct response online.

3 We preregistered 3 SDs above the *group* median, instead of the more typically used exclusion criterion of 3 SDs above the individual mean, which is what we implemented after considering that online participants would be unlikely to search for all answers. Both exclusion criteria produce the same results pattern and do not affect any statistical conclusions.

4 As this potential error is randomly distributed across participants, removing the distorted and undistorted versions of this question from Experiment 1 did not change the results.
